# Topics of Public Interest

**Published:** 1912-07

**Authors:** 


### TOPICS OF PUBLIC INTEREST

THE Child Welfare Exhibition, held in Convention Hall, Buffalo, May 27 to June 3, was most interesting, valuable, and entertaining. The illustration of the work of the municipal health department was especially to be commended. The mechanical device by which six babies revolved in view of the public while the seventh was cut off by the sickle of Death, impersonated by a grim lady doll in black was criticised as a gallery play by some but it must be remembered that it is the gallery which we are trying to reach in such popular exhibitions. The children's dances and the enlistment of the boy scouts and camp fire girls, should also be considered seriously, for a very important point in such educational work is to secure child welfare through the intelligent coöperation of the children themselves. Children are just as intelligent as adults; they sometimes lack experience and exact knowledge and usually opportunities for initiative but, beyond the first few years of life, success in their moral and physical control depends largely on training them to act for themselves. The Exhibition owed much of its success to the realization that a label without an exhibit is better than an exhibit without a label. Some mistakes were made. The teaching as to the economic and physiologic values of foods included some inaccuracies and we did not quite like the card showing how a working girl should spend her wages, how much she had to have to live, how much she got and then the question as to how she made up the deficit. The implication was a slur on a good many girls who, somehow or other, manage to accomplish the statistically impossible, without resorting to the easy and obvious way. But, as a whole, the exhibition reflected the utmost credit on its managers, the public were genuinely interested and much good will result.

There is one thought that we add with some hesitancy, as it might seem to imply a censure of many existing philanthropies although nothing is further from our intention. If we were able to take an active part in philanthropic work, we would pass by education, by schools, libraries, museums, lecture courses, &c; we would ignore the heathen, abroad and at home, as well as various strictly religious campaigns; we would establish no hospital, asylum, sanitarium or sanitary or hygienic method; we would disregard the immigrant, the blind, the insane or otherwise physically defective, the babies, the young man and woman, the aged. In short, we would try to accomplish something for the general economic, social and sanitary welfare of the average, respectable, self-supporting, middle aged man and woman. Did you ever stop to think on the dull, hard, lonesome lives of this part of the population, that appeals to us in no special way as needing guidance, sympathy, or help? Did it ever occur to you that a little effort along the same general lines so well followed for other classes, could greatly improve the comfort and increase the pleasure of living for this uninteresting class, to which, save for better opportunities and fortune, most of you and I belong? Did you ever realize how many suicides could be prevented if the victims were young enough to be reached by the Y. M. C. A. and many similar organizations, if they were blind, or crippled, or if they had some definite disease, or if they were actually senile?

---

The Columbus Hospital (Buffalo) training school held its commencement exercises June 12. Four nurses were graduated. Rev. Angelo Strazzoni, of St. Anthony's Church delivered the address and Dr. Marshall Clinton presented the diplomas. This event marked the fourth anniversary of the hospital.

---

The Buffalo General Hospital graduated a class of 18 on June 11. Capt. Calvert K. Mellen, Principal of the Lafayette High School gave an address, Dr. Wm. Ward Plummer presented diplomas and Mr. George R. Howard the badges.

---

The Lockport City Hospital held its first training school commencement June 12. Dr. C. N. Palmer delivered the address.

---

The Warsaw Hospital, Dr. W. R. Thomson resident surgeon, has issued its first annual report. In reality, the report covers only ten months of actual operation. About 170 operations are included, with a total of three deaths. Operations not done at the hospital are not included. A brief description of the technic is given and, from personal observation, we can commend both the actual work and the broad-minded spirit of the surgical staff in regard to the strictly medical features of cases.

---

The Anti-tubercular Committee of the N. Y. State Grange has issued a 20-page circular, with illustrations. In the last twenty years, the mortality from tuberculosis in cities has fallen by 23.9 deaths per hundred thousand population, while in the country, the fall has been 5.3 deaths. It is not generally known that the total rural incidence of disease and mortality is now greater than the urban. Rensselaer, Ulster, Ontario, Dutchess, Monroe and Schenectady counties now maintain tuberculosis hospitals at an average per diem, per capita cost of $1.00 to $1.40. The plans for 1912 are as follows:

A. General State-wide propaganda work.

(1) Circulation of this report in printed form.

(2) Letters to Granges urging action on various subjects from time to time.

(3) Preparation of small photographic Anti-Tuberculosis exhibits for circulation among Granges.

(4) Preparation of program for use of lecturers.

B. Intensive work to be recommended to subordinate and Pomona Granges.

(1) Possible employment of visiting nurses for the rural districts for the nursing of all cases of disease and the instruction of the public in modern methods of hygiene and sanitation.

(2) Fostering more active interest in local public health methods such as county tuberculosis hospitals, and better enforcement of the public health laws, including the law requiring the reporting of cases of tuberculosis.

(3) Efforts to improve rural public health standards through the appointment of the best possible type of town and village health officers and by eliminating political considerations entirely from these appointments and from the enforcement of public health laws.

---

The following physicians from western New York were in attendance at the recent meeting of the A. M. A.: Buffalo: Brown, Filsinger, Francis, Howe, Himmelsbach, Johnson, McKee, March, Otto, Rooth, Thornton, Ullman, Weed, Woehnert, Wilson, Bayliss, Benedict, Bennett, Congdon, Fronczac, F. P. Lewis, Love, Linklater, Potter, Rochester, Rosengren, Stockton, Townsend, Grove, Walsh, Wende, A. A. Jones; Auburn: Austin, Sears; Perry: Way; Rochester: Ballantine, Comfort, Davis, Soble, Whitbeck, Zimmer, Bowen, Mulligan, Potter, Swan, Williams, Jameson; Ithaca: Baker, Wilson, Tinker, Parker; Hornell: Barney, Kelly, Kyson; Johnstown: Beebe; Elmira: Case, Jennings, Fisher; Jamestown: Cottis, Johnson; Victor: Clapper; Canaseraga: Deegan; Rome: Gillette, Reid; Cuba: H. Gillette; Binghampton: Green, Quackenbush, Hough, Watson, Lappens, Chittenden; Danville: Gregory; Amsterdam: Hicks; Saratoga Springs: Humphrey; Watertown: W. J. Kellow, Meader; Batavia: Manchester, Johnson; Troy: Mallet; Dunkirk: Richards; Fredonia: Robson; Plattsburg: Schiff, Silver; Olean: C. Smith; Tonawanda, Britt; Middletown: Conner; Oneida: Carpenter,

Pfaff; Geneva, Knickerbocker, Skinner; East Aurora: Phelps; Ogdensburg: Madill, McIlmay; Canandaigua: McClellan.

THE SOCIETY OF MEDICINE OF PARIS, in a request for an exchange, announces that it already has more than sixty French medical journals on file and that it intends to secure the most interesting foreign journals. This establishment of a consulting library for periodic literature, is stated to be a new departure in France. This is surprising to American physicians who have free access to the important literature of the world in almost every city of any size.

The Buffalo Branch of the *American Association for the Conservation of Vision* is raising funds for this most worthy cause. The Russell Sage Foundation has promised a contribution provided that $5,000 is raised by local subscription. The local Board of Health and the Department of Education, also endorse the movement. Over 30,000 persons in the U. S. are unnecessarily blind. Dr. F. Park Lewis is President of the Buffalo Branch. Contributions may be sent to Mr. J. F. Schoellkopf, 454 Franklin St.

An emergency operation for acute inflammation of the appendix has recently been performed in the open air, at the annual encampment of Canadian troops at Niagara-on-the-Lake. The patient is recovering. This is the first major operation required during these encampments.

The Detroit and Cleveland Line has placed in commission, between Buffalo and Detroit, a new steamer costing a million and a half. In size, speed, safety, modern equipment and luxury, travel on the Great Lakes now compares favorably with ocean travel. In fact the few ocean boats that surpass the modern lake boats in size and speed are considered by veteran navigators of both salt and fresh water, to do so at the sacrifice of safety—as has recently been demonstrated.

The Corning epidemic of typhoid has given a lesson that is to be heeded. Corning and Elmira are to unite in securing a water supply from Blossburg, Pa., 50 miles distant, at a cost of a million.

Until further notice the Directory of Nurses of the Lexington Heights Hospital will be in charge of Miss Belle, 179 Franklin St., Buffalo.

The American Medical Directory contains the names of nearly 148,000 physicians, for the U. S. and Canada. The total population of the two countries is about 105,000,000—giving a ratio of about 1 physician to 700 people. While the list includes only licentiates and doubtless omits many eligible to practice but engaged in other occupations or retired which would swell the census figures, it is undoubtedly a more accurate as well as a more hopeful basis for calculating average clientele. Less than ten years ago, the average clientele was estimated at 600 or even less. The annual mortality among physicians is approximately 3,000 and the annual increment by graduation and licensure is running at about 5,000, while the annual increase of population is about 2,000,000. In other words, each graduate may either take a vacancy due to death or have a potential new clientele of 1,000. This is, by comparison, a very favorable showing but we must not be blind to the actual fact that there is still a large excess of supply over demand and must exercise every reasonable restriction on the output of fresh supply until a practical equilibrium has been reached.

---

### Government List of Industrial Poisons.

The Department of Commerce and Labor is distributing a bulletin giving for the first time the English translation of the list of industrial poisonings which the International Association for Labor Legislation through its various national organizations has been revising and completing since 1908.

Commissioner Charles P. Neil writes, "In 1910 at the time of the publication by the Bureau of Labor of the earlier list of industrial poisons little accurate information was available concerning the prevalence of cases of industrial poisoning in American factories. No state had made any legal requirement for the reporting of cases of industrial poisoning, and in the absence of such definite basis for statistical information a general impression had prevailed that American employees were largely exempt from the dangers from which industrial workers in other countries suffered."

Special investigation in the white phosphorus match industry, the inquiries of the Illinois State Commission on Occupational Diseases, and a study of the white lead and lead oxide industries disclosed 388 specific cases of lead poisoning in 16 months, and 60 deaths from lead poisoning occuring in New York in 1909 and 1910. As a result of the disclosures laws were enacted during 1911 and 1912 in eight states—California, Connecticut, Illinois, Michigan, New York, Wisconsin, Maryland and New

Jersey requiring reports by physicians of all cases of certain occupational diseases occuring in their practice.

It is expected that the American Medical Association, working in conjunction with the Association for Labor Legislation will begin a nation wide effort to stamp out the use of processes which foreign experience has shown to be as dangerous as they are unnecessary.

---

Opposition to the Establishment of an Independent Health Service.—Hon. John D. Works of California, has sent us a 78 page reprint of his speech against the Senate bill to establish a genuine public health service. His arguments are the specious ones that the A. M. A. is trying to organize a medical trust, with the existing government medical services as willing tools, and in opposition to the rights of Christian Scientists, sectarians and the public generally. It is perfectly true that the organized medical profession is trying to be its weaker and less informed brothers' keeper and it is also true that any effort in this direction, for health or anything else, by any kind of an organization, interferes with what many consider the inalienable right of the citizen to run risks for himself and others and to oppose his own opinions against those of persons better able to judge. And it is also true that the same general spirit of legislating in favor of good morals, good health and economic welfare, has not always been directed wisely or sincerely and that it has resulted in a mass fol was which, in the aggregate, make this country less free for the private individual in carrying out his own personal inclinations, than almost any other highly civilized country in the world. This fact renders any task of reform, however well conceived, difficult. We agree with Senator Works' general premises but, disagree most heartily with his application of them to this particular case.

---

Haemoglobin Examinations.—The Talqvist method of saturating filter paper and comparing with a standard is the most convenient and rapid and can be used by any one whose color vision is normal. Readings can be made with less than ten per cent. error—sufficiently accurate for clinical purposes and probably as accurate as most persons can accomplish with the more elaborate apparatus. A very important point is to compare fairly large areas of color. Modifications in which small areas are compared are liable to give misleading results. Readings should be made by day-light but not in bright sun-light. There is an old proverb not to look a gift horse in the mouth; but some of the color scales with which the profession has been presented are unreliable.

The Civil War Survivors.—According to Gen. Ainsworth's figures there were 2,128,948 men in the Union service during the Civil war, and of that number 1,726,353 were alive at the time of the grand review in 1865.

It is estimated that the survivors now number about 500,000. The mortality at present is at the rate of about 100 a day.

It is a remarkable fact that during the civil war period there were 844,801 volunteers 16 years of age or under in the Union army. But 16 was near the age limit, because there were only 1,523 who were 14 or under. Consequently nearly all the youngsters of the war period are now over 60 years of age. Far more survivors are beyond 70 than under 70.

Gen. Ainsworth estimates that by 1915 the Union veterans will number only 430,000, and only 250,000 in 1920 and only 37,000 by 1930. There are now living about 350,00 Confederate veterans. Of all those who enlisted on the one side or the other only one-fourth are survivors.

If we are correctly informed, the last two survivors of the Revolution, died within an hour of each other, in 1866, although there was some question as to the formal service of one of them.

---

The International Congress on Aerial Law, sitting in Geneva, has decided that, if a baby is born in an airship or aeroplane, it has to be registered on the ship's log or else a statement drafted concerning its birth. A copy of such statement has to be filed with the nearest public authority entitled to register births and deaths.

If the airship is flying in a foreign country a statement has to be left with the consul of the airship's nation.

---

Genesee Co. is agitating for a tuberculosis hospital.

